# Linking morphology and performance: skeletal growth and sex-specific form–function relationships in an agamid lizard

**DOI:** 10.1242/jeb.251931

**Published:** 2026-05-08

**Authors:** Subhasmita Patro, Aditya Moger, Lipsa Dash, Madhusmita Behera, Maria Thaker

**Affiliations:** ^1^ Centre for Ecological Sciences, Indian Institute of Science, Bangalore 560012, Karnataka, India; ^2^ Department of Biology, Indian Institute of Science Education and Research (IISER) Pune, Pune 411008, Maharashtra, India; ^3^ School of Biological Sciences, National Institute of Science Education and Research, Jatni 752050, Odisha, India

**Keywords:** Ontogeny, Allometry, Locomotion, Bite force, Geometric morphometrics, Reptile

## Abstract

Linking morphology to performance is essential for understanding how skeletal growth shapes functional capabilities. In sexually dimorphic species, males and females often experience distinct selection pressures, leading to differences in growth rate, allometric patterns and adult performance. Using longitudinal X-ray imaging, we quantified skeletal growth in the agamid lizard, *Psammophilus dorsalis*, from juvenile to adult stages, to examine sex differences in growth trajectories, allometric patterns, whole-body performance and morphology–performance relationships. We found that body length increased non-linearly in both sexes and growth trajectories were parallel. Key morphological traits scaled isometrically relative to body length. As adults, sexes did not differ in absolute sprint speed, but males had higher absolute bite force. Hindlimb length and body shape poorly predicted sprint speed, but head length predicted bite force in females. Overall, our results suggest broadly similar growth patterns and performance outcomes in both sexes, indicating possible constraints on morphology and performance.

## Teaser

**Summary:** Despite sex-specific selection pressures in the agamid lizard *Psammophilus dorsalis*, the sexes showed parallel growth trajectories and similar ontogenetic allometry and adult performance.

## INTRODUCTION

The relationship between form and function is fundamental to understanding fitness and the ways in which organisms interact with their environment. Morphology primarily determines performance abilities (Arnold, 1983; Irschick et al., 2008) and often shows consistent scaling patterns across species (Bonine and Garland, 1999; Isip et al., 2022). However, absolute performance capabilities can vary within a species, especially when unequal competition and predation pressure imposes distinct selection regimes on males and females (Irschick et al., 2007; Littleford-Colquhoun et al., 2019; Vanhooydonck et al., 2010). For example, in some lizard species, selection favours higher sprint speed and greater bite force in males than females (Herrel et al., 2007; 1999; Irschick and Meyers, 2007). Because performance levels are linked to key morphological traits, sex-specific selective pressures can lead to divergent patterns of association between morphology and performance in males and females, and these associations may differ across performance traits within the same species. For example, in some lizards (e.g. *Anolis carolinensis*, *Hemidactylus frenatus* and *Podarcis muralis*), males and females show different relationships between sprint speed and body size (Cameron et al., 2013; Head et al., 2024; Husak, 2006a; Simon et al., 2022; Zamora-Camacho et al., 2014). Conversely, both head size and head shape have been shown to predict bite force in both sexes in many reptilian species, with individuals that possess wider, longer or taller heads generating greater bite forces than those with shorter or flatter heads (Herrel et al., 2010; 2007; Sagonas et al., 2014; Wittorski et al., 2016). Thus, the association between morphological traits and whole-body performance can show sex-specific as well as performance-specific variation.

Sex-specific differences in adult performance can arise from differences in investment in critical skeletal traits during ontogeny (Sanger et al., 2013). Traits that confer a competitive or survival advantage, such as larger heads in males or longer trunk length in females, may experience accelerated growth due to increased allocation of resources (Bonduriansky and Day, 2003; Bonneaud et al., 2016; Kaliontzopoulou et al., 2010). The allometric slope, whether positive, negative or isometric, reflects the relative growth rate of specific body parts in relation to overall body size, thereby offering insights into differential investment, functional significance and potential selection pressures on those traits. Hence, studying ontogenetic allometry and growth rate trajectories can provide insights into the relative investment of ecologically relevant traits in males and females (Pélabon et al., 2014; Sanger et al., 2013).

Here, we examined sex-specific variation in adult performance traits, their morphological correlates and the trajectories of growth from juvenile to adult stages, in the sexually dimorphic lizard, *Psammophilus dorsalis*. Both sexes occupy similar habitats, i.e. rocky outcrops in peninsular India and feed on a diet composed largely of ants (Balakrishna et al., 2016). However, males of this species are larger, conspicuously coloured and experience greater predation risk and competition than females (Amdekar and Thaker, 2019; Batabyal and Thaker, 2017; Ranade and Isvaran, 2022). Microhabitat use and escape strategies also differ between sexes: males occupy higher rock perches, which increases their visibility and results in greater flight initiation distances when attacked (Batabyal et al., 2017). Given the inter-sexual differences in predation risk and competition, having greater sprint speed and bite force may confer greater benefits to males than to females. Because performance is closely linked to morphology, stronger selection on males for increased sprint speed and bite force may lead to stronger morphology–performance associations and positive allometry of these traits in males than females. Therefore, we predict that males and females will differ in: (a) the rate at which skeletal size and shape change during ontogenetic growth, (b) the allometric relationships of key traits with snout–vent length during ontogeny, and (c) whole-body performance (sprint speed, bite force) as adults. Given these, we also predict (d) sex-specific differences in the strength of correlation between morphology and performance in adults. To test these predictions, we tracked the skeletal growth of *P. dorsalis* from juvenile to adulthood, using X-ray imaging. At the adult stage, we measured maximum sprint speed and bite force. By integrating measurements of ontogenetic and adult morphology with functional performance, we aimed to uncover the ontogenetic basis of sex-specific performance in this species.

## MATERIALS AND METHODS

### Capture, handling and housing

Juvenile lizards (*N*=40) were captured by hand at night from rocky granite hills near Kolar, on the outskirts of Bangalore, India, between December 2022 to February 2023. Snout–vent length (SVL) was measured in the field, at the time of capture to restrict the body size range of all lizards to 63–85 mm. Lizards that were not within this size range were released immediately. At the time of capture, it was not possible for us to reliably identify the sex of the lizards. Post capture, all lizards were placed in individual cloth bags and transported to a semi-natural outdoor housing facility. Here, each lizard was placed in individual enclosures (90×50×45 cm), lined with stones for basking and shelters made of clay tiles. All lizards were provided with *ad libitum* water and mealworms and crickets, dusted alternately with calcium or vitamin powder (Zoo Med Repti Calcium, Zoo Med ReptiVite) for food daily. The lizards remained in the outdoor facility for approximately 24 weeks, until they were sexually mature adults, which is when males develop distinctive secondary sexual colouration. Throughout this period, we collected morphometric data (see below). Eleven lizards died from unknown causes during this period, of which six individuals were excluded from all analyses because their sex could not be determined. Three additional individuals were excluded from the ontogenetic morphometry and allometry analyses because of insufficient data, but were retained for the adult morphology and performance analyses.

Upon reaching sexual maturity, the animals were transferred to a dedicated lizard housing facility at the institute, where they were housed individually in glass terraria (60×30×25 cm) that were lined with paper towels and provisioned with rocks and a shelter. The housing facility was equipped with LED and UV lighting on an automated 12 h:12 h light:dark cycle and maintained at ambient temperature conditions. Individual terrariums were also set up with incandescent lamps (60 W) that were turned on for 5 h during the day to allow for basking. Lizards were provided with mealworms and grasshoppers daily for food, along with *ad libitum* water. All lizards were allowed to acclimate to laboratory conditions for 5 days before behavioural and performance trials were conducted. Lizards were housed in the lab for a total of 11 days for the duration of the experiments, after which they were chemically euthanised and preserved in 70% alcohol for future studies.

### Morphometry: 2D landmarking and geometric morphometrics

All morphometric measurements were taken 1 week after capture from the wild and subsequently, once every 3 weeks for a total duration of 21 weeks. Snout–vent length (SVL in mm) was measured using a ruler, and body mass (g) was recorded with a digital balance.

To track changes in skeletal size and shape over time, digital X-ray images were taken using a portable X-ray machine (Alerio Neo dental X-ray machine paired with PZ Medical's DR detector). The X-ray was set at 70 kV and 0.2 s exposure time, positioned at a fixed height of 1 m from the surface of the detector. Lizards (*N*=31) were temporarily immobilized by immersing them in crushed ice for 3 min before imaging. All X-ray images were cropped, sharpened and adjusted for contrast in the PZ DICOM image viewer and converted from the original DICOM format to .jpeg format for further processing. X-ray images were scaled and landmarked using the tpsDig232 and tpsUtil32 software by three different people. Every image contained 59, 2-dimesional landmarks (Fig. S1), selected based on their consistent presence in all images and their reliability in providing a clear and repeatable summary of skeletal morphology. The landmarks represent 2-dimensional cartesian co-ordinates of the contact points between bones, joints or tips of processes. To account for user error, all images were landmarked in replicates of three by the same people in random order. For the final analysis, landmarks were averaged across all three replicates. Using the averaged landmarks, we obtained linear measurements (Euclidean distance) of various body parts, specifically head length, head width, forelimb (humerus+ulna), 4th digit of forelimb, hindlimb (femur+tibia), metatarsus, 4th digit of hindlimb and inter-limb length, using the *geomorph* package (https://CRAN.R-project.org/package=geomorph) in R (r-project.org). Skeletal wireframe obtained from a landmarked and averaged 2D X-ray image using *MorphoJ* (Klingenberg, 2011) is shown in Fig. S2A.

We quantified four distinct sets of shape measures in this study: (i) head shape across ontogeny (day 0–day 126), (ii) body shape across ontogeny (day 0–day 126), (iii) adult head shape (between days 130–140), and (iv) adult body shape (between days 130–140). To quantify shape, we removed all landmarks corresponding to the fore- and hindlimbs, which project outside the body as changes in limb position on the X-ray do not represent the true shape variation in the lizards. We retained landmarks only on the head and main body (Fig. S2B, S2C) and performed a Generalized Procrustes Analyses (GPA) using *geomorph* package, to translate, rotate and scale the landmarked coordinates corresponding to each image to a common reference frame, ensuring that only shape data, independent of size, was retained. Subsequently, we conducted a principal component analysis (PCA) on the Procrustes-aligned shape coordinates, using *geomorph*, to identify the major axes of morphological variation within each dataset.

### Performance measures: bite force and sprint speed

All performance measures were recorded on adult lizards (males: *N*=14; females: *N*=15), after they attained sexual maturity. We measured bite force following the protocol outlined by Amdekar and Thaker (2022). In brief, lizards were induced to bite on the stainless-steel plates of a custom-made bite force meter, with a miniature load cell (25 lbf, WMC Sealed Stainless Steel Mini Load Cell, Interface, USA) sandwiched between the plates. Each lizard was allowed to bite on the plates three times in sequence, with 1 min of rest between the trials. The highest bite force (in Newtons) recorded across the three trials was considered the maximum bite force. One male with an unusually high bite force (18.42 N) was excluded from the analyses, as it represented an outlier in the dataset.

To measure the maximum sprint speed, individual lizards were made to run on a 3.7 m racetrack that was marked at 25 cm intervals. This racetrack was lined with fine-grained sandpaper on the floor and smooth acrylic walls. The width of the track was narrow (12 cm) to restrict the movement of the lizards to only one direction during a run. At one end of the track, a shelter was placed so that the lizards would be motivated to run towards it. A typical run was initiated by releasing the lizards on one end of the track and then gently tapping their tail with a blunt flat-ended stick. All lizards were made to run the entire length of the track twice, with approximately 10 s of rest between each trial. Each lizard completed the trial in several short bouts rather than a single continuous end-to-end run. The entire run sequence was recorded using a GoPro8 camera mounted above the racetrack. To quantify the distance travelled during each bout, we used *Tracker* software (v. 6.3.2; https://opensourcephysics.github.io/tracker-online/), in which we positioned a digital tracker between the lizard's hindlimbs to record its position at the beginning and end of each running bout. Displacement was calculated as the difference between these two positions and sprint speed for each bout was estimated by dividing the displacement by the time elapsed between the two measurements. We applied a minimum displacement threshold of 20 cm, such that any running bout that covered less than this distance was excluded from sprint speed calculations. The highest sprint speed recorded across all running bouts from both the trials was considered the maximum sprint speed. One female lizard with exceptionally low sprint speed (0.48 ms^−1^) was excluded from subsequent analyses, as its low speed likely reflected a lack of motivation rather than true performance capacity.

Ethics clearance and protocols for this project were approved by the institute animal ethics committee (CAF/Ethics/865/2021). Collection permits for *P. dorsalis* were not required during field studies as it is not protected under the Schedules of the Indian Wildlife (Protection) Act, 1972.

### Statistical analyses

#### Ontogenetic changes in morphometry and allometry

To examine ontogenetic changes in body size, we fitted a mixed-effect quadratic model, with SVL as the response variable, sex (categorical) and time (continuous) and their interaction as predictors, and individual ID as a random factor [*lme4* (https://CRAN.R-project.org/package=lme4) and *lmerTest* (https://CRAN.R-project.org/package=lmerTest)]. Model comparison using Akaike Information Criterion (AIC) indicated that the quadratic model provided a better fit than the linear model (AIC_linear_=1180, AIC_quadratic_=1166, Chisq=18.5, *P*<0.01). To investigate ontogenetic changes in head and body shape, we fitted separate Procrustes ANOVAs using Procrustes shape coordinates of head shape or body shape as the response variable, with sex, time and their interaction as predictors. Individual ID was included as a repeated-measures factor (*geomorph*). To visualise how head and body shape change across ontogeny, we performed a PCA on the pooled Procrustes shape coordinates for both males and females, from all the time points using the *geomorph* package. Variation in head shape was summarized by PC1 (28.28%) and PC2 (22.77%), and variation in body shape by PC1 (37.24%) and PC2 (23.44%).

To examine ontogenetic shifts in allometric relationships, we fitted separate standardised regression models (SMA) for males and females with log transformed morphometric traits as the predictor and log (SVL) as the response at every time point [*Smatr* (https://cran.r-project.org/package=Smatr); Warton et al., 2012]. The slope (*b*) obtained from the regression model at each time point was tested against the expectation of isometric growth (slope H0: 1) using 95% confidence intervals of the slope obtained from the regression model and *P*-value of *F*-statistics from a separate one-sample test of SMA slope.

#### Morphology and performance measures of adults

All variables were log-transformed before analysis to meet the assumptions of parametric analyses. We then tested for multicollinearity among key morphometric traits in adult lizards. Head length and head width were not strongly correlated to each other (males: *R*=0.5, females: *R*=0.65). Hindlimb length (femur+tibia) was strongly correlated with most other morphometric traits (*R*>0.70; Fig. S3), whereas forelimb length (humerus+ulna) exhibited less consistent associations. Together, these measures capture the overall variation in limb morphology. Thus, we retained the forelimb length, hindlimb length, head length and head width for all subsequent analyses of allometry and performance. We excluded measurements of the toes of both fore- and hindlimbs from all analyses because they were bent or folded in some images leading to unreliable measures.

Absolute sprint speed and bite force measures of adult males and females was compared using Welch's two sample *t*-test, since both bite force and sprint speed were normally distributed (Shapiro–Wilk's test). Log-transformed performance and size-related morphometric measures were then individually regressed against log(SVL) to obtain size-corrected residuals. PC1 and PC2 of shape variables in all models/analyses were retained as is, without any transformation or size-correction.

To test the effect of adult head size and shape on bite force, we first summarized the head shape by performing a PCA on the Procrustes shape coordinates of only the head region for all adult lizards. PC1 and PC2 obtained from this analysis explained 36.14% and 25.22% of the variation, respectively. We then fitted separate linear regression models for males and females with bite force as the response and head length, head width, PC1 and PC2 of the adult head shape as predictors (*lme4*). Two sets of models were constructed: one using log-transformed values and another using size-corrected values.

Similarly, to test the effect of adult limb lengths and body shape on sprint speed, we first summarized body shape by conducting a PCA on the Procrustes shape coordinates of the entire body (excluding head and limbs) for all adult lizards (*geomorph*). PC1 and PC2 obtained from this analysis explained 40.78% and 32.55% of the variation, respectively. We then fitted separate linear regression models for males and females with sprint speed as the response variable and forelimb length, hindlimb length and PC1 and PC2 of the adult body shape as predictors. Two sets of models were constructed: one using log-transformed values and another using size-corrected values. We also performed pairwise Pearson's correlation tests to examine overall associations between sprint speed and the rest of the morphometric traits using both log-transformed values and size-corrected residual measures.

All statistical analyses were performed in RStudio (v. 4.5.1; r-project.org).

## RESULTS AND DISCUSSION

### Ontogenetic changes in morphometry and allometry

**Fig. 1.**
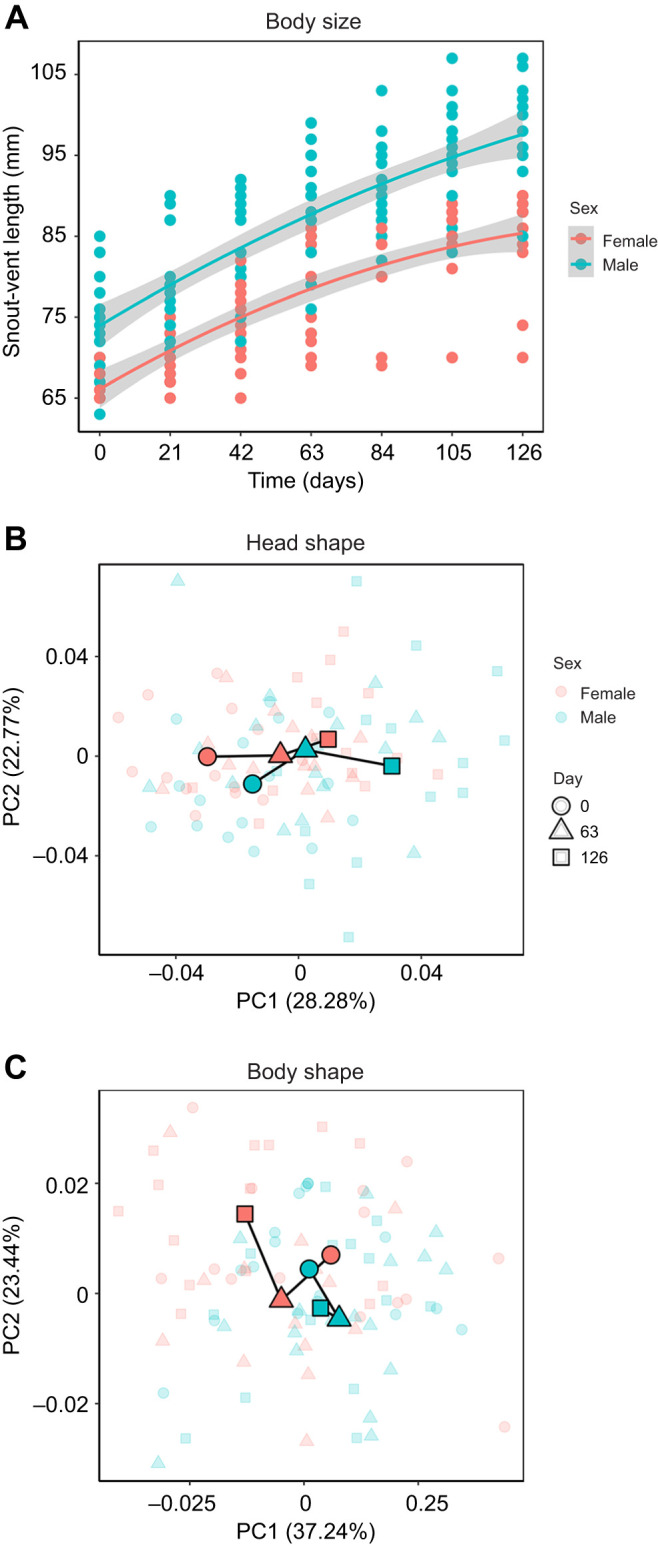
**Ontogenetic changes in body size (snout–vent length), head shape and body shape in *Psammophilus dorsalis* from juvenile to adult stages.** (A) Quadratic regression plots depicting parallel growth trajectories for both sexes (shaded areas: 95% CI). Principal component analysis (PCA) of (B) head shape and (C) body shape reveals sex-specific growth trajectories. Three time points are shown here, with mean values highlighted by outlined and saturated symbols. (*N*=14 females, 17 males at day 0). See Table S1 for detailed statistics.

Males and females often experience different selection pressures owing to distinct reproductive strategies, which can lead to differences in growth rate, morphology, and performance. In *P. dorsalis*, body size (SVL) increased non-linearly over time in both sexes (time: β=5.65, *P*<0.01; time²: β=−0.31, *P*<0.01; Fig. 1A; Table S1). SVL was influenced independently by both time (*t*=8.25, *P*<0.01) and sex (*t*=3.2, *P*<0.01), with males being on average 7.93 mm larger than females throughout ontogeny (Fig. 1A). This difference was already evident at the time of capture, when juvenile males were on average 7.41 mm larger than juvenile females, indicating that sexual size dimorphism is established early. The interaction term sex×time or sex×time^2^ had no significant effect on SVL (sex×time: *t*=−0.17, *P*=0.86; sex×time^2^: *t*=1.02, *P*=0.3; Table S1), indicating that both males and females had parallel growth trajectories. Together, the fixed effects of time and sex explained 68.27% of the variance in SVL (marginal *R*^2^), which increased to 92.55% after including individual ID as a random effect (conditional *R*^2^). This early dimorphism and parallel growth trajectories resemble patterns observed in other lizard genera such as *Anolis*, *Lacertids* and *Podarcis* (Kaliontzopoulou et al., 2008; Sanger et al., 2013; Seifan et al., 2009).

Ontogenetic changes in head and body shape were significantly influenced by the interaction of sex and time (head: *F*=1.07, *P*<0.01; body: *F*=1.5, *P*=0.01; Table S1). Head shape, in many lizards is linked to signalling and competition and is thus proposed to be maintained by sexual selection (Lopez-Darias et al., 2015; Scharf and Meiri, 2013). In *P. dorsalis*, both males and females perform head-bobbing displays during social interactions with conspecifics (Batabyal and Thaker, 2019; Patro et al., 2025; Ranade et al., 2022 preprint), yet males experience greater competition than females, owing to overlapping territories (Ranade and Isvaran, 2022). Thus, we expected the head shape trajectories to diverge between sexes. However, PCA plots for visual representation show that head shape changed similarly in both sexes, becoming longer and narrower over time (Fig. 1B). In contrast, body shape trajectories diverged between sexes: males developed wider bodies, whereas females became progressively narrower over time (Fig. 1C). These differences in body shape may reflect sex-specific ecological and reproductive demands. Since male *P. dorsalis* maintain and defend larger territories than females (Ranade and Isvaran, 2022), differences in space use may favour body shapes that facilitate greater locomotion and territorial defence in males. On the other hand, female body shape may be more strongly influenced by fecundity selection associated with egg storage (Olsson et al., 2002; Scharf and Meiri, 2013).

**Fig. 2.**
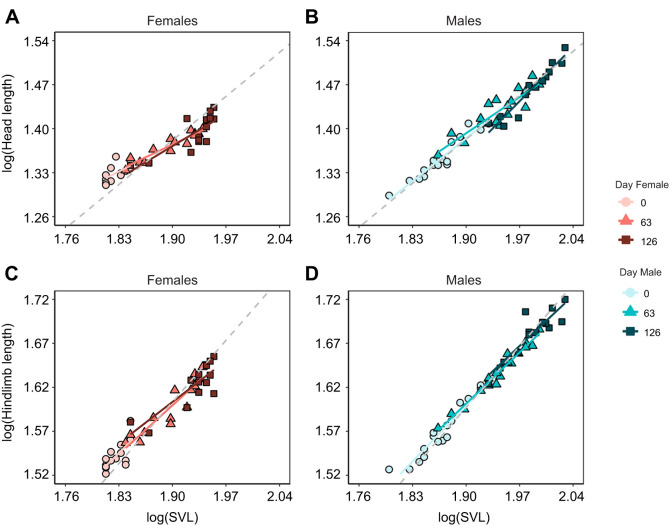
**Regression lines depicting the allometric relationships of head and hindlimb length (mm) in *P. dorsalis* at three time points during ontogenetic growth.** The grey dashed line represents the slope for perfect isometry (H0=1) (*N*=14 females, 17 males at day 0). See Table S2 for detailed statistics for these and other morphological traits.

Studies in vertebrates suggest that performance-related traits can exhibit strong allometric scaling (Herrel and Gibb, 2006, but see Voje, 2016). Given that male *P. dorsalis* experience higher competition and predation, we predicted a shift towards positive allometry in head size and limb lengths during ontogeny, as these traits might contribute to increased male survival and reproductive success (Kaliontzopoulou et al., 2008). However, this prediction was not supported by our results. Between days 0 and 126, the allometric slopes (*b*) of head length, head width, forelimb length, and hindlimb length relative to body size (SVL) did not differ consistently or significantly from isometry in either sex (*P*>0.05), with the 95% confidence intervals overlapping 1 (Fig. 2; Table S2). Thus, all morphological traits scaled largely isometrically with body size during ontogeny, indicating proportional growth and no evidence of differential investment between the sexes. Contrary to our results, a multi-generational study on a different population of *P. dorsalis* found positive allometry in male head width, which was influenced by environmental stress and competition (Valiya Parambil and Isvaran, 2025). Thus, allometric patterns are likely context dependent and can vary depending on the temporal scale of measurement and ecological conditions.

### Morphology and performance measures of adults

**Fig. 3.**
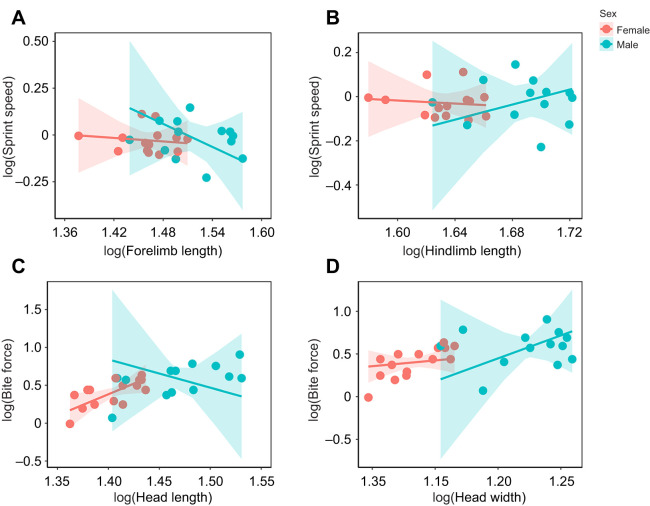
**Regression plots showing the relationships between morphology and performance in adult *P. dorsalis* (shaded areas represent 95% confidence intervals).** Maximum sprint speed was not significantly influenced (*P*>0.05) by either (A) forelimb length or (B) hindlimb length in males (*N*=13) or females (*N*=14) (measured in mm). (C) Bite force (N) was positively influenced by head length (mm) in females but not males. (D) However, head width (mm) did not influence bite force in either males (*N*=13) or females (*N*=15). All traits were log transformed prior to analysis.

In lizards, territorial conflicts are often resolved through displays or assessment behaviours, but can sometimes escalate to physical contests involving biting, where bite force can influence contest outcomes (Baird et al., 2013; Huyghe et al., 2005; López and Martín, 2001). In *P. dorsalis*, we expected males to have higher bite force, since they are larger and face stronger intra-specific competition owing to overlapping territories with other males during the mating season (Ranade and Isvaran, 2022). Approximately 15% of the male–male interactions in *P. dorsalis* escalate to physical attacks involving biting (Batabyal and Thaker, 2019; Patro et al., 2025). Additionally, since head size is known to signal dominance in some lizards (Bush et al., 2016; Huyghe et al., 2005), a positive correlation between head size and bite force may be expected. Consistent with these predictions, adult males in our study exhibited greater absolute bite force than females (Welch two sample *t*-test: *t*=2.6, *P*=0.02; Fig. S4B). However, a positive relationship between uncorrected bite force and head length was detected only in females (*P*<0.05; Table S3A, Fig. 3C), suggesting that head morphology may play a stronger role in determining bite performance in females. After controlling for body size, residual head length and width had no effect on bite force in both sexes (*P*>0.05; Fig. S5; Table S3B), indicating that further increases in head dimensions beyond what is expected by body size does not provide additional performance benefits. This indicates that the influence of morphology on bite force in females operates primarily through their overall body size. The positive relationship observed in female lizards in our study is consistent with findings from some reptilian taxa that have reported correlations between head length and bite force (Deeming, 2022; Isip et al., 2022), although this pattern is not universal and different studies have identified other aspects of head morphology, including jaw musculature, as important predictors of bite force. Although head shape is also known to influence bite force in lizards (Herrel et al., 2001a,b), the association between head shape and bite force in our study was weak (*P*>0.05; Table S3B). In some lizard species, diet plays a role in shaping jaw and bite mechanics, thereby influencing bite force (Herrel et al., 2001a,b; Saulnier Masson et al., 2023). However, because males and females of *P. dorsalis* consume similar diets, predominantly comprising ants, diet is unlikely to explain the observed sex differences in bite force (Balakrishna et al., 2016).

Sprint speed is an ecologically critical trait that can influence an animal's ability to escape predators, and chase opponents. Hence, maximum sprint speed may be under positive selection, particularly in conspicuous animals, such as male *P. dorsalis* (Amdekar and Thaker, 2019). In our study, absolute maximum sprint speed did not differ between the sexes (Welch two sample *t*-test: *t*=0.37, *P*=0.71; Fig. S4A), despite clear dimorphism in body size and shape. These results contradict previous findings from wild-caught *P. dorsalis*, wherein males were found to be faster than females (Batabyal et al., 2017), potentially owing to differences in motivation between lab-reared and wild-caught lizards. Although longer hindlimb length is linked to greater stride length and locomotor power (Goodman et al., 2008; Lowie et al., 2019), several studies across multiple lizard genera report weak correlation between sprint speed and hindlimb length (Clemente et al., 2009; Feiner et al., 2020; Garland, 1985). In *P. dorsalis*, maximum sprint speed, both before and after size correction, was not influenced by forelimb length, hindlimb length or body shape in either sex (*P*>0.05; Fig. 3; Fig. S5; Table S3), consistent with findings reported by Amdekar and Thaker (2022). Similarly, sprint speed showed no significant correlations with the rest of the limb-related morphometric traits in either males or females (*P*>0.05; Table S4). These findings imply that different morphologies can yield comparable locomotor outcomes, potentially through compensatory mechanisms such as enhanced muscle or joint mechanics (Clemente et al., 2009; Higham et al., 2011). Moreover, Husak (2006b) suggested that actual field escape speeds may be more ecologically relevant than maximum sprint speeds measured in laboratories, as they are more likely to be under direct selection. Ecological factors such as motivation or perceived risk may also influence sprint performance, resulting in a decoupling of locomotor ability from skeletal morphology.

In sum, despite clear sexual dimorphism in *Psammophilus dorsalis* and prior evidence indicating sex-specific differences in competition, predation risk and space use, both sexes show similar sprint performance and underlying morphological patterns. Such similarities suggest possible mechanistic constraints and compensatory processes shaping sprint performance. However, bite performance and its morphological correlates differed between sexes, suggesting potentially distinct selection pressures acting on them. Overall, growth rates and relative investment in most body parts were largely similar between the sexes, except for the sex-specific differences in body shape, suggesting conserved underlying developmental mechanisms.

## Supplementary Material

10.1242/jexbio.251931_sup1 Supplementary information

Dataset 1 contains all raw data used in the analyses presented in the manuscript, “*Patro et al. 2026 - Linking morphology and performance: skeletal growth and sex-specific form-function relationships in an agamid lizard.*” The file includes three sheets: **Sheet 1: Metadata -** Descriptions of all column headings used in the datasets. **Sheet 2: Skeletal Growth Data -** Morphometric data collected from P. dorsalis across multiple time points, from juvenile to adult stages. **Sheet 3: Adult Morphology and Performance -** Morphometric and performance data measured in adult P. dorsalis.

## References

[JEB251931C1] Amdekar, M. S. and Thaker, M. (2019). Risk of social colours in an agamid lizard: implications for the evolution of dynamic signals. *Biol. Lett.* 15, 20190207. 10.1098/rsbl.2019.0207 31088284 PMC6548737

[JEB251931C2] Amdekar, M. S. and Thaker, M. (2022). Colours of stress in male Indian rock agamas predict testosterone levels but not performance. *Horm. Behav.* 144, 105214. 10.1016/j.yhbeh.2022.105214 35696781

[JEB251931C3] Arnold, S. J. (1983). Morphology, Performance and Fitness. *Am. Zool.* 23, 347-361. 10.1093/icb/23.2.347

[JEB251931C4] Baird, T. A., Hardy, I. C. W. and Briffa, M. (2013). Lizards and other reptiles as model systems for the study of contest behaviour. In *Animal Contests* (ed. I. C. W. Hardy and M. Briffa), pp. 258-286. Cambridge University Press. 10.1017/CBO9781139051248.014

[JEB251931C5] Balakrishna, S., Batabyal, A. and Thaker, M. (2016). Dining in the City: Dietary Shifts in Indian Rock Agamas across an Urban–Rural Landscape. *J. Herpetol.* 50, 423-428. 10.1670/14-073

[JEB251931C6] Batabyal, A. and Thaker, M. (2017). Signalling with physiological colours: high contrast for courtship but speed for competition. *Anim. Behav.* 129, 229-236. 10.1016/j.anbehav.2017.05.018

[JEB251931C7] Batabyal, A. and Thaker, M. (2019). Social coping styles of lizards are reactive and not proactive in urban areas. *Gen. Comp. Endocrinol.* 270, 67-74. 10.1016/j.ygcen.2018.10.007 30336119

[JEB251931C8] Batabyal, A., Balakrishna, S. and Thaker, M. (2017). A multivariate approach to understanding shifts in escape strategies of urban lizards. *Behav. Ecol. Sociobiol.* 71, 83. 10.1007/s00265-017-2307-3

[JEB251931C9] Bonduriansky, R. and Day, T. (2003). The evolution of static allometry in sexually selected traits. *Evolution* 57, 2450-2458. 10.1111/j.0014-3820.2003.tb01490.x 14686522

[JEB251931C10] Bonine, K. E. and Garland, T. (1999). Sprint performance of phrynosomatid lizards, measured on a high-speed treadmill, correlates with hindlimb length. *J. Zool.* 248, 255-265. 10.1111/j.1469-7998.1999.tb01201.x

[JEB251931C11] Bonneaud, C., Marnocha, E., Herrel, A., Vanhooydonck, B., Irschick, D. J. and Smith, T. B. (2016). Developmental plasticity affects sexual size dimorphism in an anole lizard. *Funct. Ecol.* 30, 235-243. 10.1111/1365-2435.12468

[JEB251931C12] Bush, J. M., Quinn, M. M., Balreira, E. C. and Johnson, M. A. (2016). How do lizards determine dominance? Applying ranking algorithms to animal social behaviour. *Anim. Behav.* 118, 65-74. 10.1016/j.anbehav.2016.04.026

[JEB251931C13] Cameron, S. F., Wynn, M. L. and Wilson, R. S. (2013). Sex-specific trade-offs and compensatory mechanisms: bite force and sprint speed pose conflicting demands on the design of geckos (*Hemidactylus frenatus*). *J. Exp. Biol.* 216, 3781-3789. 10.1242/jeb.083063 23821718

[JEB251931C14] Clemente, C. J., Thompson, G. G. and Withers, P. C. (2009). Evolutionary relationships of sprint speed in Australian varanid lizards. *J. Zool.* 278, 270-280. 10.1111/j.1469-7998.2009.00559.x

[JEB251931C15] Deeming, D. C. (2022). Inter-relationships among body mass, body dimensions, jaw musculature and bite force in reptiles. *J. Zool.* 318, 23-33. 10.1111/jzo.12981

[JEB251931C16] Feiner, N., Munch, K. L., Jackson, I. S. C. and Uller, T. (2020). Enhanced locomotor performance on familiar surfaces is uncoupled from morphological plasticity in *Anolis* lizards. *J. Exp. Zool. A Ecol. Integr. Physiol.* 333, 284-294. 10.1002/jez.2349 31994351

[JEB251931C17] Garland, T. (1985). Ontogenetic and individual variation in size, shape and speed in the Australian agamid lizard *Amphibolurus nuchalis*. *J. Zool.* 207, 425-439. 10.1111/j.1469-7998.1985.tb04941.x

[JEB251931C18] Goodman, B. A., Miles, D. B. and Schwarzkopf, L. (2008). Life on the rocks: habitat use drives morphological and performance evolution in lizards. *Ecology* 89, 3462-3471. 10.1890/07-2093.1 19137951

[JEB251931C19] Head, A., Vaughn, P. L., Livingston, E. H., Colwell, C., Muñoz, M. M. and Gangloff, E. J. (2024). Include the females: morphology–performance relationships vary between sexes in lizards. *J. Exp. Biol.* 227, jeb248014. 10.1242/jeb.248014 39155657

[JEB251931C20] Herrel, A. and Gibb, A. C. (2006). Ontogeny of Performance in Vertebrates. *Physiol. Biochem. Zool.* 79, 1-6. 10.1086/498196 16380923

[JEB251931C21] Herrel, A., Spithoven, L., Van Damme, R. and De Vree, F. (1999). Sexual dimorphism of head size in *Gallotia galloti*: testing the niche divergence hypothesis by functional analyses. *Funct. Ecol.* 13, 289-297. 10.1046/j.1365-2435.1999.00305.x

[JEB251931C22] Herrel, A., Damme, R. V., Vanhooydonck, B. and Vree, F. D. (2001a). The implications of bite performance for diet in two species of lacertid lizards. *Can. J. Zool.* 79, 662-670. 10.1139/z01-031

[JEB251931C23] Herrel, A., De Grauw, E. and Lemos-Espinal, J. A. (2001b). Head shape and bite performance in xenosaurid lizards. *J. Exp. Zool.* 290, 101-107. 10.1002/jez.1039 11471139

[JEB251931C24] Herrel, A., Mcbrayer, L. D. and Larson, P. M. (2007). Functional basis for sexual differences in bite force in the lizard *Anolis carolinensis*. *Biol. J. Linn. Soc.* 91, 111-119. 10.1111/j.1095-8312.2007.00772.x

[JEB251931C25] Herrel, A., Moore, J. A., Bredeweg, E. M. and Nelson, N. J. (2010). Sexual dimorphism, body size, bite force and male mating success in tuatara. *Biol. J. Linn. Soc.* 100, 287-292. 10.1111/j.1095-8312.2010.01433.x

[JEB251931C26] Higham, T. E., Korchari, P. G. and McBrayer, L. D. (2011). How muscles define maximum running performance in lizards: an analysis using swing- and stance-phase muscles. *J. Exp. Biol.* 214, 1685-1691. 10.1242/jeb.051045 21525314

[JEB251931C27] Husak, J. F. (2006a). Does speed help you survive? A test with collared lizards of different ages. *Funct. Ecol.* 20, 174-179. 10.1111/j.1365-2435.2006.01069.x

[JEB251931C28] Husak, J. F. (2006b). Does survival depend on how fast you can run or how fast you do run? *Funct. Ecol.* 20, 1080-1086. 10.1111/j.1365-2435.2006.01195.x

[JEB251931C29] Huyghe, K., Vanhooydonck, B., Scheers, H., Molina-Borja, M. and Van Damme, R. (2005). Morphology, performance and fighting capacity in male lizards, *Gallotia galloti*. *Funct. Ecol.* 19, 800-807. 10.1111/j.1365-2435.2005.01038.x

[JEB251931C30] Irschick, D. J. and Meyers, J. J. (2007). An analysis of the relative roles of plasticity and natural selection in the morphology and performance of a lizard (*Urosaurus ornatus*). *Oecologia* 153, 489-499. 10.1007/s00442-007-0726-y 17453255

[JEB251931C31] Irschick, D., Bailey, J. K., Schweitzer, J. A., Husak, J. F. and Meyers, J. J. (2007). New directions for studying selection in nature: studies of performance and communities. *Physiol. Biochem. Zool.* 80, 557-567. 10.1086/521203 17909993

[JEB251931C32] Irschick, D. J., Meyers, J. J., Husak, J. F. and Le Galliard, J. (2008). How does selection operate on whole-organism functional performance capacities? A review and synthesis. *Evol. Ecol. Res.* 10, 177-197. 10.7275/R58G8HX6

[JEB251931C33] Isip, J. E., Jones, M. E. H. and Cooper, N. (2022). Clade-wide variation in bite-force performance is determined primarily by size, not ecology. *Proc. R. Soc. B* 289, 20212493. 10.1098/rspb.2021.2493 PMC8864353 35193399

[JEB251931C34] Kaliontzopoulou, A., Carretero, M. A. and Llorente, G. A. (2008). Head shape allometry and proximate causes of head sexual dimorphism in *Podarcis* lizards: joining linear and geometric morphometrics. *Biol. J. Linn. Soc.* 93, 111-124. 10.1111/j.1095-8312.2007.00921.x

[JEB251931C35] Kaliontzopoulou, A., Carretero, M. A. and Llorente, G. A. (2010). Intraspecific ecomorphological variation: linear and geometric morphometrics reveal habitat-related patterns within *Podarcis bocagei* wall lizards. *J. Evol. Biol.* 23, 1234-1244. 10.1111/j.1420-9101.2010.01984.x 20406345

[JEB251931C36] Klingenberg, C. P. (2011). MorphoJ: an integrated software package for geometric morphometrics. *Mol. Ecol. Res.* 11, 353-357. 10.1111/j.1755-0998.2010.02924.x 21429143

[JEB251931C37] Littleford-Colquhoun, B. L., Clemente, C., Thompson, G., Cristescu, R. H., Peterson, N., Strickland, K., Stuart-Fox, D. and Frere, C. H. (2019). How sexual and natural selection shape sexual size dimorphism: Evidence from multiple evolutionary scales. *Funct. Ecol.* 33, 1446-1458. 10.1111/1365-2435.13337

[JEB251931C38] López, P. and Martín, J. (2001). Fighting rules and rival recognition reduce costs of aggression in male lizards, *Podarcis hispanica*. *Behav. Ecol. Sociobiol.* 49, 111-116. 10.1007/s002650000288

[JEB251931C39] Lopez-Darias, M., Vanhooydonck, B., Cornette, R. and Herrel, A. (2015). Sex-specific differences in ecomorphological relationships in lizards of the genus *Gallotia*. *Funct. Ecol.* 29, 506-514. 10.1111/1365-2435.12353

[JEB251931C40] Lowie, A., Gillet, E., Vanhooydonck, B., Irschick, D. J., Losos, J. B. and Herrel, A. (2019). Do the relationships between hind limb anatomy and sprint speed variation differ between sexes in *Anolis* lizards? *J. Exp. Biol.* 222, jeb188805. 10.1242/jeb.188805 30683664

[JEB251931C41] Olsson, M., Shine, R., Wapstra, E., Ujvari, B. and Madsen, T. (2002). Sexual dimorphism in lizard body shape: the roles of sexual selection and fecundity selection. *Evolution* 56, 1538-1542. 10.1111/j.0014-3820.2002.tb01464.x 12206252

[JEB251931C42] Patro, S., Saravanan, T., Parag, A. and Thaker, M. (2025). Dialogues in colour and behaviour: integration of complex signalling traits and physiology. *Proc. R. Soc. B* 292, 20250118. 10.1098/rspb.2025.0118 PMC12187413 40555365

[JEB251931C43] Pélabon, C., Firmat, C., Bolstad, G. H., Voje, K. L., Houle, D., Cassara, J., Rouzic, A. L. and Hansen, T. F. (2014). Evolution of morphological allometry. *Ann. N. Y. Acad. Sci.* 1320, 58-75. 10.1111/nyas.12470 24913643

[JEB251931C45] Ranade, D. and Isvaran, K. (2022). Inferring Social Interactions Over a Lifespan from Space-Use Patterns in a Tropical Agamid. *J. Herpetol.* 56, 164-171. 10.1670/20-068

[JEB251931C46] Ranade, D., Karatgi, R., Mahishi, S. and Isvaran, K. (2022). Competing females strategically combine signals and physical aggression differently from males in a polygynous tropical lizard. *bioRxiv*, 2022-2009. 10.1101/2022.09.27.508996

[JEB251931C47] Sagonas, K., Pafilis, P., Lymberakis, P., Donihue, C. M., Herrel, A. and Valakos, E. D. (2014). Insularity affects head morphology, bite force and diet in a Mediterranean lizard: Head morphology in *Lacerta trilineata*. *Biol. J. Linn. Soc.* 112, 469-484. 10.1111/bij.12290

[JEB251931C48] Sanger, T. J., Sherratt, E., McGlothlin, J. W., Brodie, E. D. III, Losos, J. B. and Abzhanov, A. (2013). Convergent evolution of sexual dimorphism in skull shape using distinct developmental strategies. *Evolution* 67, 2180-2193. 10.1111/evo.12100 23888844

[JEB251931C49] Saulnier Masson, R., Daoues, K., Measey, J. and Herrel, A. (2023). The evolution of bite force and head morphology in scincid lizards: diet and habitat use as possible drivers. *Biol. J. Linn. Soc.* 140, 58-73. 10.1093/biolinnean/blad052

[JEB251931C50] Scharf, I. and Meiri, S. (2013). Sexual dimorphism of heads and abdomens: Different approaches to ‘being large’ in female and male lizards: Sexual Dimorphism in Lizards. *Biol. J. Linn. Soc.* 110, 665-673. 10.1111/bij.12147

[JEB251931C51] Seifan, M., Gilad, A., Klass, K. and Werner, Y. L. (2009). Ontogenetically stable dimorphism in a lacertid lizard (*Acanthodactylus boskianus*) with tests of methodology and comments on life-history. *Biol. J. Linn. Soc.* 97, 275-288. 10.1111/j.1095-8312.2009.01200.x

[JEB251931C52] Simon, M. N., Cespedes, A. M. and Lailvaux, S. P. (2022). Sex-specific multivariate morphology/performance relationships in *Anolis carolinensis*. *J. Exp. Biol.* 225, jeb243471. 10.1242/jeb.243471 35363299

[JEB251931C53] Valiya Parambil, G. and Isvaran, K. (2025). Costly traits in a dynamic world: trait responses to fine-scale varying environment differ according to selection pressures in a tropical lizard. *Evolution* 79, 681-697. 10.1093/evolut/qpaf018 39883057

[JEB251931C54] Vanhooydonck, B., Cruz, F. B., Abdala, C. S., Azócar, D. L. M., Bonino, M. F. and Herrel, A. (2010). Sex-specific evolution of bite performance in *Liolaemus* lizards (Iguania: Liolaemidae): the battle of the sexes. *Biol. J. Linn. Soc.* 101, 461-475. 10.1111/j.1095-8312.2010.01519.x

[JEB251931C58] Voje, K. L. (2016). Scaling of morphological characters across trait type, sex, and environment: a meta-analysis of static allometries. *Am. Nat.* 187, 89-98. 10.1086/684159 27277405

[JEB251931C55] Warton, D. I., Duursma, R. A., Falster, D. S. and Taskinen, S. (2012). . smatr 3–an R package for estimation and inference about allometric lines. *Method. Ecol. Evol.* 3, 257-259. 10.1111/j.2041-210X.2011.00153.x

[JEB251931C56] Wittorski, A., Losos, J. B. and Herrel, A. (2016). Proximate determinants of bite force in *Anolis* lizards. *J. Anat.* 228, 85-95. 10.1111/joa.12394 26471984 PMC4694155

[JEB251931C57] Zamora-Camacho, F. J., Reguera, S., Rubiño-Hispán, M. V. and Moreno-Rueda, G. (2014). Effects of limb length, body mass, gender, gravidity, and elevation on escape speed in the lizard *Psammodromus algirus*. *Evol. Biol.* 41, 509-517. 10.1007/s11692-014-9285-4

