# Supplementary material for: Linking morphology and performance: skeletal growth and sex-specific form–function relationships in an agamid lizard
Source: J Exp Biol. 2026 May 8;229(9):jeb251931. doi: 10.1242/jeb.251931 (PMC13200722; doi:10.1242/jeb.251931)
Supplement: Supplementary information [file jexbio-229-251931-s1.pdf]

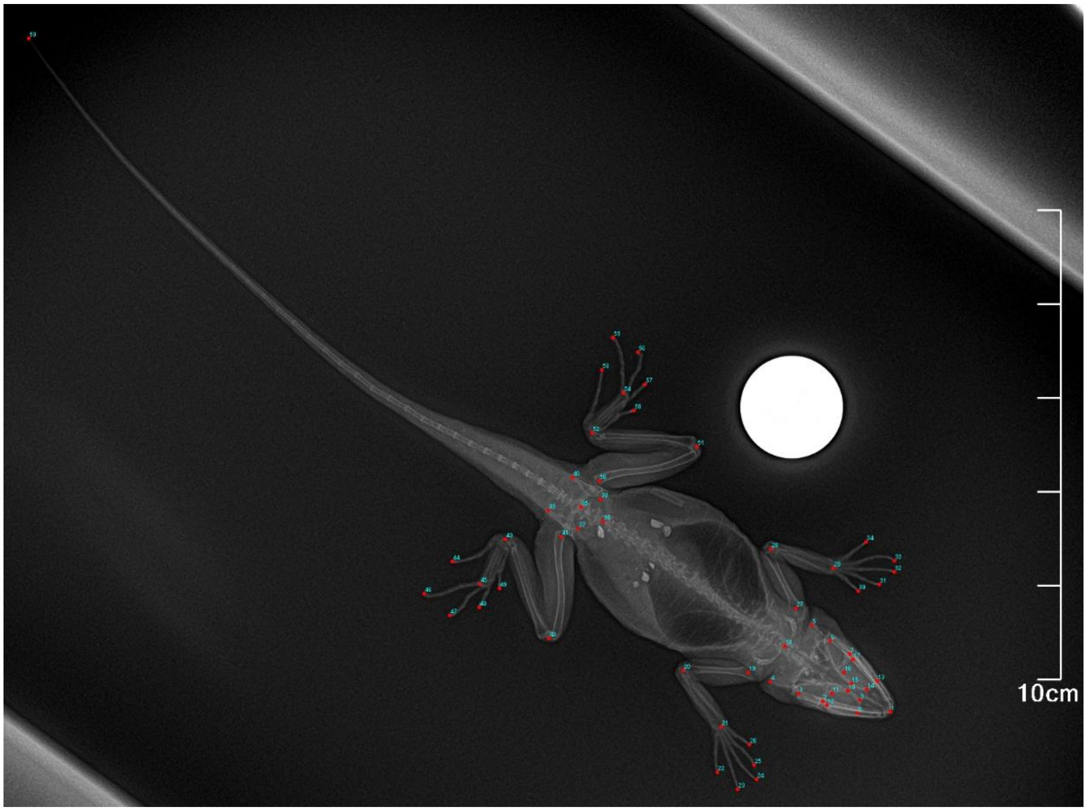

Fig. S1. Landmarked 2-dimensional X-ray image of *Psammophilus dorsalis*. The metal coin in the image, was used to verify the internally generated scale reference in all images.

A **B** **C**

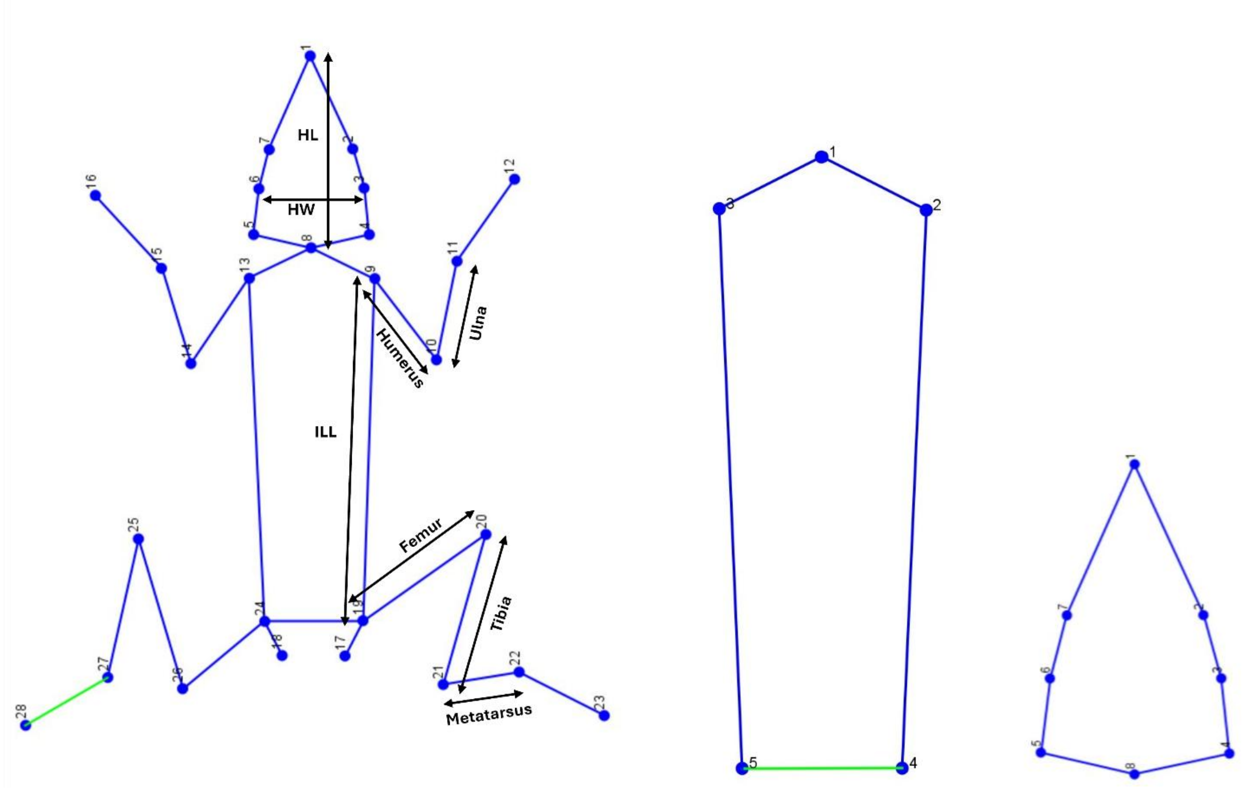

Fig. S2. Wireframe of the landmarked X-ray images of *P. dorsalis*. A) Complete skeletal frame of *P. dorsalis* illustrating the morphological traits measured in this study. HL = Head length, HW = Head width, ILL = Inter-limb length, Humerus + Ulna = Forelimb length and Femur + Tibia = Hindlimb length. B) Skeletal frame used in all body shape analyses. C) Skeletal frame used in all head shape analyses. Note: Unused landmarks were removed from the landmarked image of Figure S1 to obtain the above wireframes.

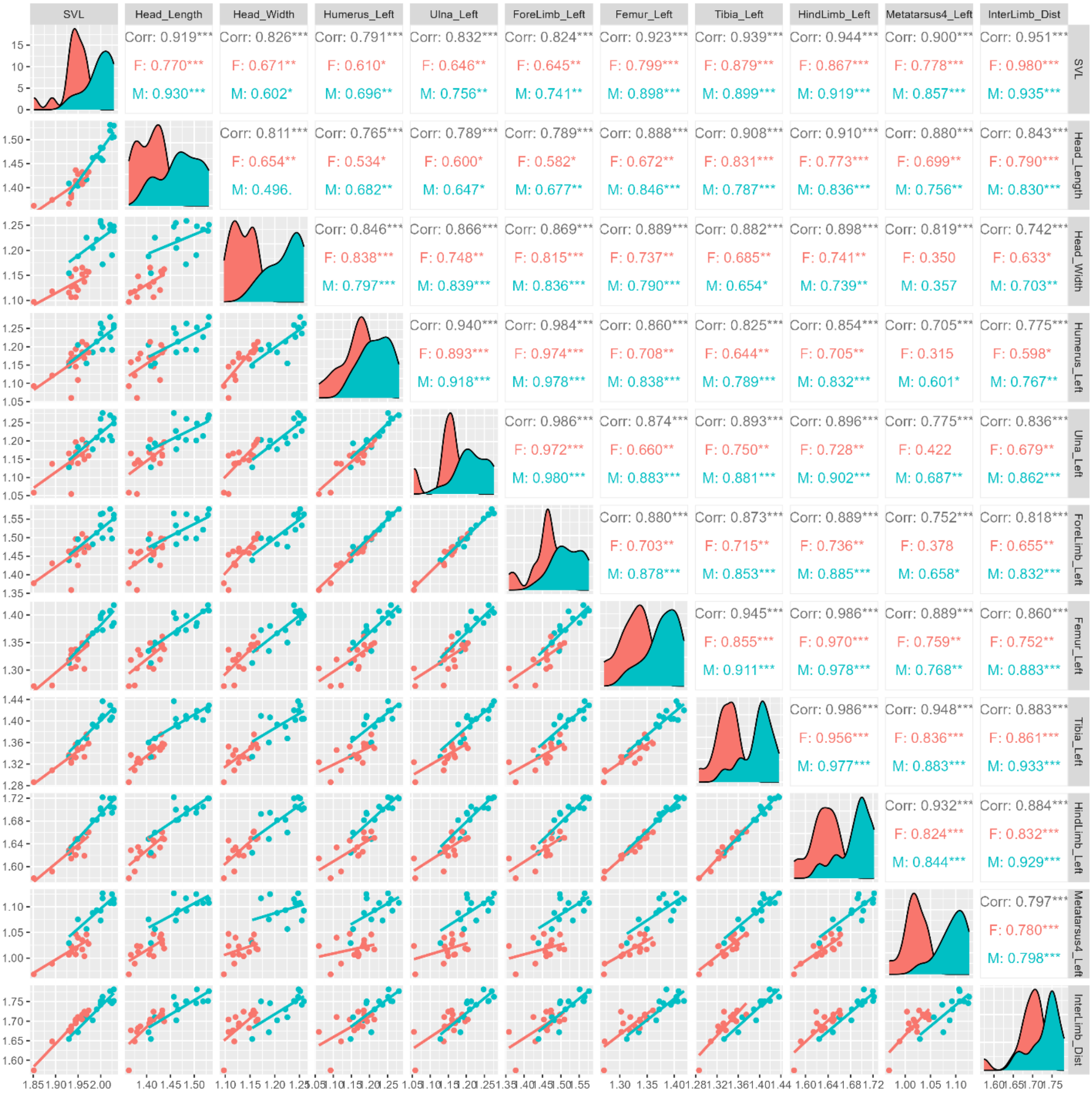

Fig. S3. Correlation matrix showing the strength of correlations between different morphological traits in adult male (green) and female (red) *P. dorsalis*. Scatter plots are shown in the lower left triangle, and Pearson's correlation coefficients with significance levels (*) are presented in the upper right triangle. Overall correlation coefficient for both sexes combined are shown in black. All traits were log-transformed before analyses.

Journal of Experimental Biology • Supplementary information

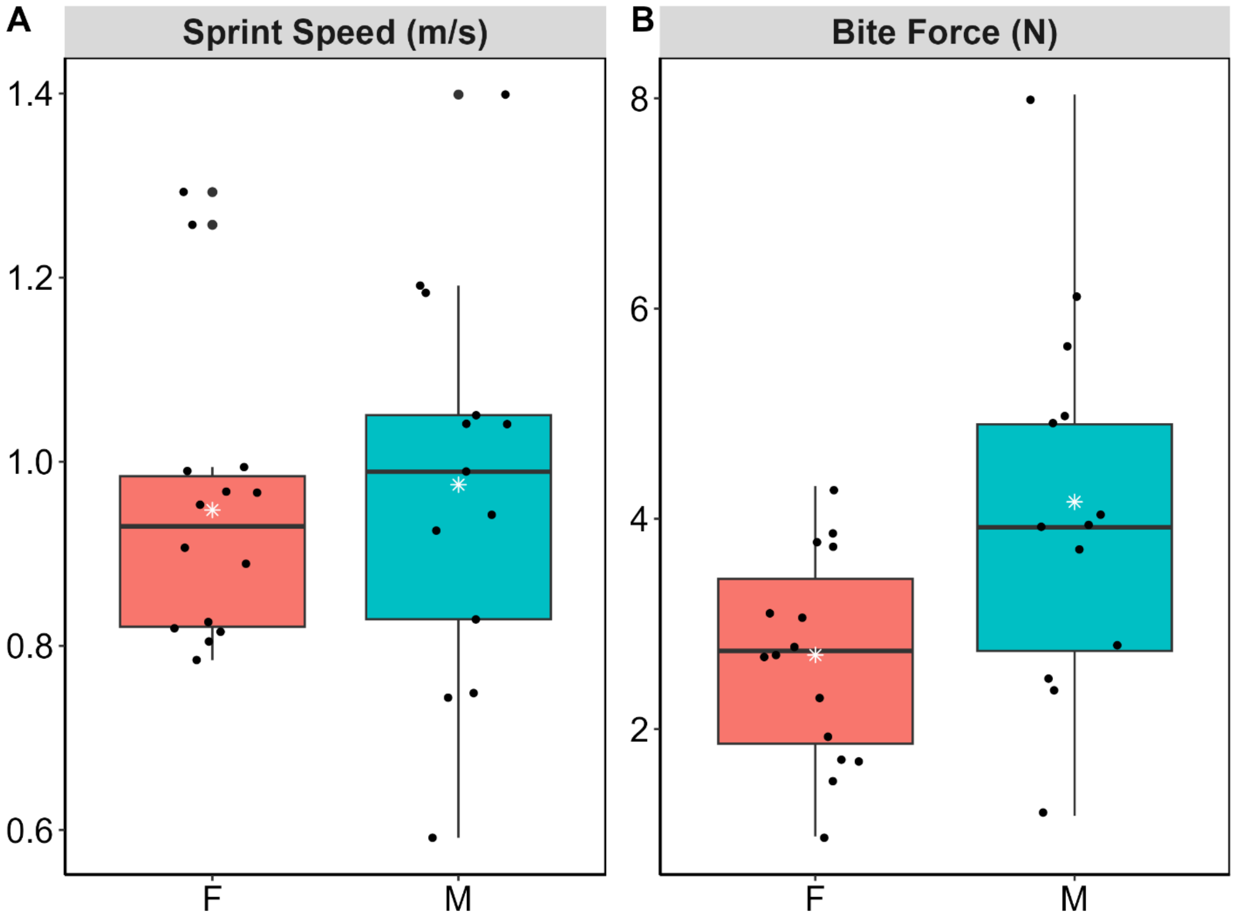

Fig. S4. Maximum sprint speed and bite force in adult *P. dorsalis*. A) Sexes show no significant difference in sprint speed (Welch two sample t-test: t = 0.37, p = 0.71; N = 14 females, 13 males), but B) differ in bite force (Welch two sample t-test: t = 2.6, p = 0.02; N = 15 females, 13 males). F = females, M = males. Shown are boxplots with the mean (*), median, first (Q1) and third quartiles (Q3) of the raw values (before log transformation).

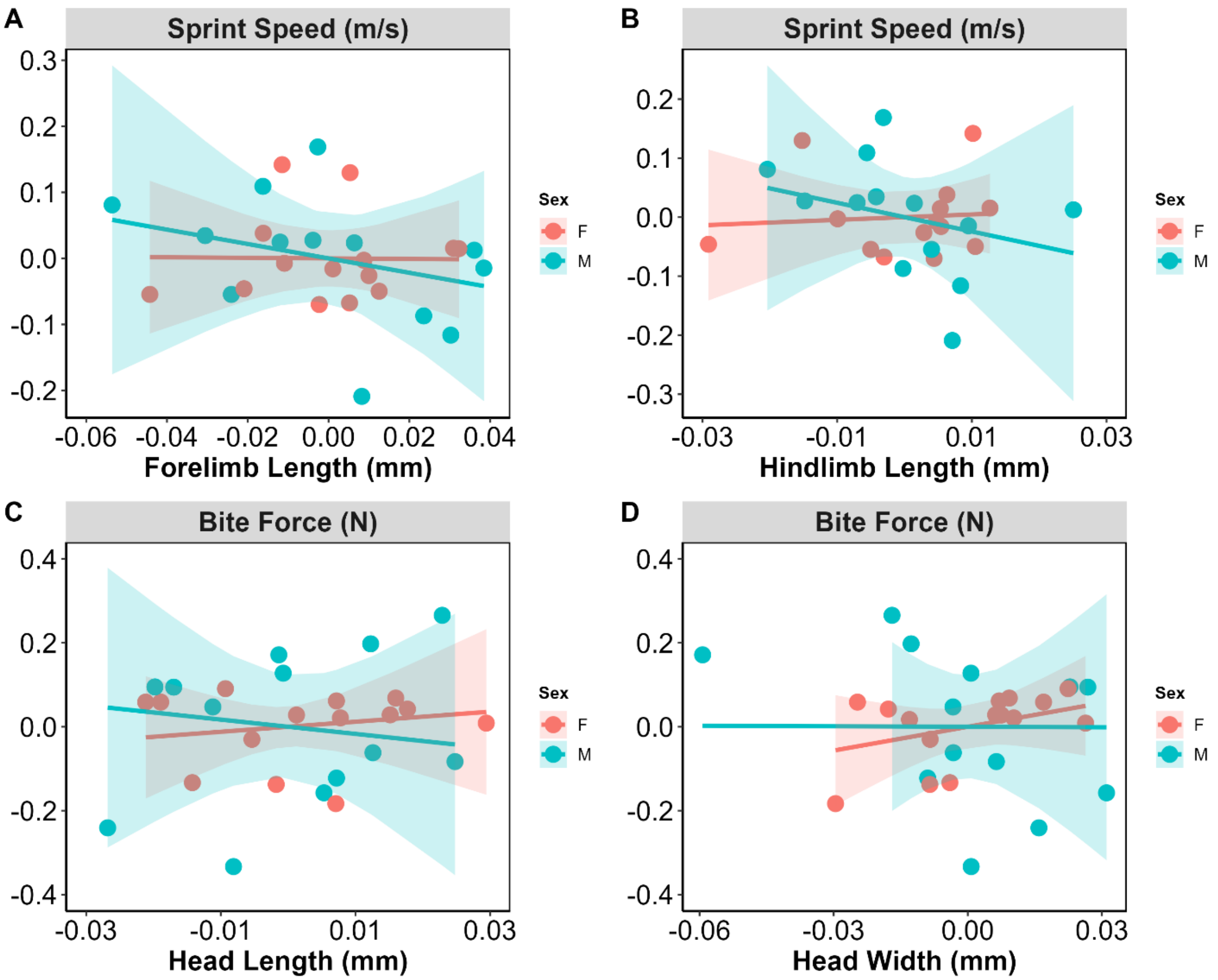

Fig. S5. Regression plots depicting the association between residual morphology and performance in adult *P. dorsalis* (shaded areas indicate 95% confidence intervals). Maximum sprint speed was not significantly influenced ($p > 0.05$) by either A) forelimb length or B) hindlimb length in both males (N = 13) and females (N = 14). Similarly, bite force was not significantly influenced by either C) head length or D) head width in both males (N = 13) and females (N = 15). All traits were log transformed and size-corrected before analyses.

Dataset 1 contains all raw data used in the analyses presented in the manuscript, *"Patro et al. 2026 – Linking morphology and performance: skeletal growth and sex-specific form–function relationships in an agamid lizard."* The file includes three sheets:

Sheet 1: Metadata – Descriptions of all column headings used in the datasets.

Sheet 2: Skeletal Growth Data – Morphometric data collected from *P. dorsalis* across multiple time points, from juvenile to adult stages.

Sheet 3: Adult Morphology and Performance – Morphometric and performance data measured in adult *P. dorsalis*.

Available for download at
https://journals.biologists.com/jeb/article-lookup/doi/10.1242/jeb.251931#supplementary-data

Table S1. The effects of time and sex on body size, head shape and body shape during ontogeny, from juvenile to adult stage, in *Psammophilus dorsalis*. Shown are i) model coefficients for the linear and quadratic terms of a mixed-effects model, their standard errors, and the associated p-values from t-tests for body size (snout to vent length), and ii) the proportion of variance explained (Rsq), F-statistics, effect sizes and the associated p-values derived from Procrustes ANOVA for head and body shape. * indicates significant values at p < 0.05.

Response	Predictors	*estimate*	*SE*	*t*	*p-value*
	**Time**	5.65	0.69	8.25	< 0.01*
	**Time^2**	-0.31	0.08	-3.65	< 0.01*
**Body Size (SVL)**	**SexM**	7.93	2.47	3.2	< 0.01*
	**Time:SexM**	-0.16	0.93	-0.17	0.87
	**Time^2:SexM**	0.12	0.11	1.02	0.31
		Rsq	F	Z	*p-value*
	**Time**	0.12	4.88	8.67	< 0.01*
**Head Shape**	**SexM**	0.05	13.29	12.5	< 0.01*
	**Time:SexM**	0.03	1.07	2.65	< 0.01*
	**Time**	0.04	1.64	2.55	< 0.01*
**Body Shape**	**SexM**	0.05	10.36	3.83	< 0.01*
	**Time:SexM**	0.04	1.5	2.42	0.01*

Table S2. Allometry of morphological traits relative to SVL across ontogeny. Allometric slope (*b*), 95% confidence interval of slope (*b*) and p-value of the F-statistic comparing *b* with H0: 1 (isometry), for multiple morphological traits in *P. dorsalis* across ontogeny from juvenile to adult stage. * indicates significant values at p < 0.05.

		Head Length			Head Width			Fore Limb Length			Hind Limb Length		
	**Time (days)**	*Slope b*	*95% CI*	*p-value*	*Slope b*	*95% CI*	*p-value*	*Slope b*	*95% CI*	*p-value*	*Slope b*	*95% CI*	*p-value*
**Males**	0	1.01	0.87-1.17	0.87	0.87	0.69-1.09	0.21	1.11	0.84-1.46	0.45	0.93	0.81-1.08	0.33
	21	0.97	0.76-1.23	0.73	0.97	0.74-1.26	0.8	1.11	0.77-1.59	0.57	0.99	0.81-1.22	0.94
	42	1	0.83-1.20	0.98	0.93	0.7-1.22	0.57	1.06	0.79-1.42	0.68	0.94	0.83-1.06	0.28
	63	0.92	0.72-1.18	0.5	0.88	0.62-1.25	0.45	1.01	0.8-1.29	0.91	0.84	0.77-0.93	< 0.01*
	84	1.07	0.85-1.33	0.56	0.93	0.61-1.4	0.71	1.35	1.03-1.77	0.03*	0.95	0.85-1.10	0.6
	105	1.14	0.87-1.50	0.34	1.02	0.7-1.47	0.92	1.16	0.75-1.79	0.5	0.91	0.76-1.10	0.32
	126	1.29	1.08-1.54	< 0.01*	1.08	0.65-1.8	0.75	1.04	0.66-1.65	0.85	0.91	0.69-1.18	0.44
**Females**	0	1.01	0.63-1.63	0.96	1.25	0.73-2.16	0.4	1.86	1.11-3.13	0.02*	1.23	0.80-1.89	0.33
	21	1.22	0.78-1.90	0.36	0.66	0.38-1.16	0.14	1.25	0.71-2.21	0.43	1.03	0.71-1.48	0.88
	42	0.94	0.69-1.27	0.66	0.83	0.56-1.24	0.35	0.97	0.62-1.52	0.89	0.9	0.72-1.12	0.32
	63	0.61	0.50-0.75	< 0.01*	0.68	0.44-1.04	0.08	0.79	0.55-1.13	0.18	0.86	0.68-1.08	0.17
	84	0.63	0.44-0.90	0.01*	0.75	0.51-1.1	0.13	1.1	0.71-1.7	0.64	0.82	0.62-1.09	0.15
	105	0.81	0.57-1.16	0.23	0.81	0.56-1.18	0.25	1.1	0.76-1.6	0.59	0.8	0.60-1.07	0.12
	126	0.93	0.63-1.32	0.62	0.78	0.49-1.24	0.28	1.15	0.67-1.97	0.6	0.8	0.57-1.1	0.17

		Humerus Length			Ulna Length			Femur Length			Tibia Length			4th Digit Metatarsus Length		
	**Time (days)**	*Slope b*	*95% CI*	*p-value*	*Slope b*	*95% CI*	*p-value*	*Slope b*	*95% CI*	*p-value*	*Slope b*	*95% CI*	*p-value*	*Slope b*	*95% CI*	*p-value*
**Males**	0	1.1	0.82-1.48	0.5	1.15	0.87-1.53	0.31	0.98	0.84-1.13	0.72	0.91	0.77-1.08	0.25	0.92	0.72-1.17	0.48
	21	1.12	0.75-1.68	0.56	1.14	0.82-1.6	0.41	1.28	0.95-1.72	0.09	0.84	0.69-1.02	0.07.	2.21	1.44-3.37	< 0.01*
	42	0.97	0.72-1.29	0.8	1.21	0.87-1.66	0.24	0.98	0.82-1.17	0.81	0.92	0.81-1.04	0.18	0.95	0.75-1.21	0.67
	63	0.85	0.67-1.09	0.19	1.2	0.93-1.56	0.15	0.9	0.78-1.05	0.18	0.82	0.72-0.93	< 0.01*	0.88	0.69-1.13	0.3
	84	1.37	1.02-1.84	0.04*	1.39	1.05-1.84	0.02*	1.04	0.91-1.18	0.58	0.92	0.78-1.09	0.32	1.04	0.75-1.45	0.8
	105	1.26	0.82-1.93	0.29	1.11	0.71-1.75	0.63	1.01	0.83-1.23	0.92	0.85	0.69-1.05	0.12	0.93	0.71-1.23	0.6
	126	1.05	0.65-1.69	0.84	1.12	0.7-1.78	0.63	0.94	0.72-1.22	0.6	0.9	0.67-1.21	0.47	0.91	0.67-1.23	0.51
**Females**	0	2	1.15-3.47	0.02*	1.9	1.17-3.11	0.01*	1.35	0.86-2.11	0.18	1.16	0.75-1.78	0.48	1.76	1.14-2.71	0.01*
	21	-1.04	-1.87--0.57	0.9	1.77	1.05-3	0.03*	1.03	0.71-1.5	0.86	1.09	0.73-1.61	0.65	1.33	0.97-1.82	0.07.
	42	0.92	0.56-1.54	0.75	1.1	0.73-1.65	0.64	0.95	0.7-1.29	0.73	0.89	0.74-1.07	0.21	1.03	0.77-1.38	0.8
	63	0.7	0.45-1.08	0.1	0.95	0.67-1.34	0.76	0.86	0.65-1.15	0.28	0.87	0.71-1.08	0.19	0.93	0.72-1.21	0.58
	84	1.1	0.68-1.77	0.68	1.17	0.78-1.77	0.42	0.89	0.65-1.21	0.43	0.77	0.6-1.01	0.06.	0.83	0.6-1.16	0.26
	105	1.06	0.7-1.6	0.77	1.23	0.84-1.8	0.26	0.9	0.65-1.27	0.54	0.74	0.56-0.99	0.04*	0.76	0.55-1.06	0.1
	126	1.14	0.66-1.98	0.62	1.26	0.74-2.16	0.38	0.9	0.61-1.33	0.57	0.75	0.55-1.02	0.07.	0.77	0.53-1.1	0.14

Table S3. The effect of morphological traits, head shape (PC1 and PC2) and body shape (PC1 and PC2) on performance measures in adults of *P. dorsalis*. Table A reports results from linear regression models using log-transformed variables, whereas Table B presents results from models fitted using size-corrected residual values. Shown are model coefficients from the linear regression analyses, standard errors, and associated p-values from t-tests.

A

Response	Sex	Predictors	*estimate*	*SE*	*t*	*p-value*
**Sprint Speed**	Males	**Forelimb Length**	-2.05	1.9	-1.08	0.31
	(N=13)	**Hindlimb Length**	1.7	2.54	0.67	0.52
		**PC1_BodyShape**	0.77	1.93	0.4	0.7
		**PC2_BodyShape**	1.45	2.57	0.57	0.59
	Females	**Forelimb Length**	-0.31	1.02	-0.31	0.77
	(N=14)	**Hindlimb Length**	-0.34	1.4	-0.25	0.81
		**PC1_BodyShape**	3.52	2.53	1.39	0.2
		**PC2_BodyShape**	-0.87	2.25	-0.39	0.71
**Bite Force**	Males	**Head Length**	-3.73	5.96	-0.63	0.55
	(N=13)	**Head Width**	5.38	5.79	0.93	0.38
		**PC1_HeadShape**	-6.51	4.98	-1.31	0.23
		**PC2_HeadShape**	-1.44	2.56	-0.57	0.59
	Females	**Head Length**	5.6	2.41	2.32	0.04*
	(N=15)	**Head Width**	1.35	2.22	0.61	0.56
		**PC1_HeadShape**	2.02	2.23	0.9	0.39
		**PC2_HeadShape**	4.26	1.74	2.46	0.03*

B

Response	Sex	Predictors	*estimate*	*SE*	*t*	*p-value*
**Sprint Speed**	Males	**Forelimb Length**	-1.09	1.81	-0.6	0.56
	(N=13)	**Hindlimb Length**	-2.43	4.17	-0.58	0.58
		**PC1_BodyShape**	0.86	1.72	0.5	0.63
		**PC2_BodyShape**	0.36	1.92	0.19	0.86
	Females	**Forelimb Length**	-0.04	1.07	-0.04	0.97
	(N=14)	**Hindlimb Length**	0.46	1.83	0.25	0.81
		**PC1_BodyShape**	3.13	2.5	1.25	0.24
		**PC2_BodyShape**	-0.59	2.31	-0.26	0.8
**Bite Force**	Males	**Head Length**	-1.7	5.01	-0.34	0.74
	(N=13)	**Head Width**	-0.04	4.08	-0.01	0.99
		**PC1_HeadShape**	-2.5	2.88	-0.87	0.41
		**PC2_HeadShape**	-2.18	2.72	-0.8	0.45
	Females	**Head Length**	1.2	2.92	0.41	0.69
	(N=15)	**Head Width**	1.89	1.85	1.02	0.33
		**PC1_HeadShape**	0.88	1.8	0.49	0.64
		**PC2_HeadShape**	1.48	1.98	0.75	0.47

Journal of Experimental Biology • Supplementary information

Table S4. Relationships between performance measures and limb-related morphological traits not used in the linear model. Pearson's correlation coefficients (r) and associated p-values describing relationships between, A) log-transformed sprint speed and morphological traits, and B) residual of log-transformed sprint speed and morphological traits in adult *P. dorsalis*. * indicates significant values at p < 0.05.

**A**		**Female**		**Male**	
**Performance Trait**	**Morphological Trait**	*Pearson's r*	*p-value*	*Pearson's r*	*p-value*
**Sprint Speed**	Humerus	-0.16	0.59	-0.27	0.37
	Ulna	0.01	0.96	-0.07	0.83
	Femur	-0.12	0.67	-0.07	0.83
	Tibia	-0.09	0.76	-0.02	0.95
	Metatarsus 4th digit	-0.11	0.71	0.22	0.47
	Inter-limb Length	-0.17	0.57	0.1	0.74

**B**		**Female**		**Male**	
**Performance Trait**	**Morphological Trait**	*Pearson's r*	*p-value*	*Pearson's r*	*p-value*
**Sprint Speed**	Humerus	-0.05	0.87	-0.52	0.07
	Ulna	0.25	0.4	-0.28	0.35
	Femur	0.03	0.93	-0.42	0.15
	Tibia	0.14	0.63	-0.36	0.23
	Metatarsus 4th digit	0.04	0.88	0.2	0.52
	Inter-limb Length	0.02	0.96	-0.08	0.79

Journal of Experimental Biology • Supplementary information
